# Meteorological Report for July, 1879

**Published:** 1879-08

**Authors:** 


### METEOROLOGICAL REPORT FOR JULY, 1879.

| RAIN. DATE | RAIN. DAY. | Mean daily Barometer. | Mean daily Thermometer. | Mean daily Humidity. | Maximum Thermometer | Minimum Thermometer | Prevailing direction. |
|---|---|---|---|---|---|---|---|
| 1 | 0 | 30.100 | 78.5 | 52.0 | 84 | 69 | E. |
| 2 | 0 | 30 178 | 76 2 | 50.0 | 85 | 65 | E. |
| 3 | 0 | 30.128 | 78 5 | 44.7 | 84 | 67 | E. |
| 4 | 0 | 30.103 | 82.2 | 41.7 | 89 | 68 | S. E. |
| 5 | 0 | 30.003 | 85.7 | 50.3 | 91 | 73 | W. |
| 6 | 0 | 30.008 | 78 7 | 65 3 | 90 | 71 | N. E. |
| 7 | 0 | 30.034 | 81 5 | 63.0 | 90 | 70 | E. |
| 8 | 0 | 30 024 | 86.2 | 57.0 | 93 | 73 | W. |
| 9 | 0 | 29.950 | 87.7 | 48.3 | 94 | 78 | W. |
| 10 | 0 | 29.879 | 89.2 | 35.5 | 95 | 81 | W. |
| 11 | 0 | 29.795 | 91 2 | 44.0 | 97 | 81 | N. W. |
| 12 | 0 | 29.753 | 92 2 | 42 0 | 97 | 80 | W. |
| 13 | 0 | 29 834 | 86.5 | 48.7 | 97 | 79 | W. |
| 14 | 03 | 29 947. | 75.5 | 79 7 | 85 | 72 | N. E. |
| 15 | 0 | 29 980 | 82 0 | 67.7 | 87 | 71 | E. & S. |
| 16 | 0 | 29 951 | 85.0 | 59.3 | 91 | 72 | S. |
| 17 | 0 | 29 880 | 85.0 | 61 3 | 92 | 74 | S. |
| 18 | 87 | 29.911 | 75.2 | 81.7 | 87 | 73 | S. W. |
| 19 | 40 | 29.978 | 69.0 | 79.7 | 73 | 67 | E. |
| 20 | 0 | 30.051 | 74.5 | 51.7 | 81 | 65 | E. |
| 21 | 0 | 30.099 | 74.2 | 57.7 | 81 | 68 | E. |
| 22 | 0 | 29.990 | 78.7 | 58.7 | 84 | 68 | E. |
| 23 | 14 | 29.886 | 78.5 | 73.7 | 85 | 72 | W. |
| 24 | 0 | 29.857 | 83.7 | 63.3 | 90 | 73 | S. W. |
| 25 | 40 | 29.869 | 78 7 | 77.7 | 83 | 75 | S. |
| 26 | 1 06 | 29.860 | 77.5 | 82.3 | 86 | 73 | S. W. |
| 27 | 1.27 | 29.924 | 75.5 | 88.7 | 82 | 73 | S. W. |
| 28 | 23 | 29.951 | 78.5 | 77.0 | 86 | 74 | S. W. |
| 29 | 91 | 29.966 | 73 2 | 90.0 | 80 | 73 | S. W. |
| 30 | 18 | 30.011 | 72 5 | 88.3 | 76 | 71 | S. W. |
| 31 | 26 | 30.104 | 73.0 | 86 0 | 82 | 70 | E. |
| Monthly Means. | | 29.968 | 80.14 | 63.2 | 87.0 | 72.2 | East. |

Highest Barometer, 30.246 on the 2d.
Lowest Barometer, 29.706, on the 12th.
Monthly range of Barometer, 0.540.
Highest Temp. 97°, on 11th, 12th, 13th. Lowest Temp. 65°, on 2d, 20th.
Monthly range of Temperature, 32°.
Greatest daily range of Temperature, 21°, on the 4th.
Least daily range of Temperature, 5°, on the 30th.
Mean of Maximum Temperatures, 87°0.
Mean of Minimum Temperatures, 72°2.
Mean daily range of Temperature, 14°8. Total rainfall, 5.75 inches.
Prevailing wind, East. Total movement of wind, 56.08 miles.
Maximum velocity of wind and direction, 24 miles per hour, Southwest and East, on the 17th and 19th.
*Number of Foggy days, none. No. Cloudy days on which rain fell, 10.
Number of cloudy days on which no rain fell, 2.
Total number of days on which rain fell, 12.

H. HALL, Corp'l. Signal Service U.S.

Atlanta, Ga., July 1, 1879.

